# Anti-Inflammatory Effects of Fargesin on Chemically Induced Inflammatory Bowel Disease in Mice

**DOI:** 10.3390/molecules23061380

**Published:** 2018-06-07

**Authors:** Bei Yue, Yi-Jing Ren, Jing-Jing Zhang, Xiao-Ping Luo, Zhi-Lun Yu, Gai-Yan Ren, A-Ning Sun, Chao Deng, Zheng-Tao Wang, Wei Dou

**Affiliations:** Shanghai Key Laboratory of Formulated Chinese Medicines, Institute of Chinese Materia Medica, Shanghai University of Traditional Chinese Medicine, Shanghai 201203, China; YBrunning@163.com (B.Y.); renyijing0526@126.com (Y.-J.R.); zhangjingjing8818@163.com (J.-J.Z.); luoxpcq@163.com (X.-P.L.); yuzhilun688@sina.com (Z.-L.Y.); rengaiyan@126.com (G.-Y.R.); sunaning2009@126.com (A.-N.S.); kimi-deng@163.com (C.D.)

**Keywords:** inflammatory bowel disease, dextran sulfate sodium, NF-κB, fargesin

## Abstract

Fargesin is a bioactive lignan from *Flos Magnoliae*, an herb widely used in the treatment of allergic rhinitis, sinusitis, and headache in Asia. We sought to investigate whether fargesin ameliorates experimental inflammatory bowel disease (IBD) in mice. Oral administration of fargesin significantly attenuated the symptoms of dextran sulfate sodium (DSS)-induced colitis in mice by decreasing the inflammatory infiltration and myeloperoxidase (MPO) activity, reducing tumor necrosis factor (TNF)-α secretion, and inhibiting nitric oxide (NO) production in colitis mice. The degradation of inhibitory κBα (IκBα), phosphorylation of p65, and mRNA expression of nuclear factor κB (NF-κB) target genes were inhibited by fargesin treatment in the colon of the colitis mice. In vitro, fargesin blocked the nuclear translocation of p-p65, downregulated the protein levels of inducible NO synthase (iNOS) and cyclooxygenase-2 (COX-2), and dose-dependently inhibited the activity of NF-κB-luciferase in lipopolysaccharide (LPS)-stimulated RAW264.7 macrophages. Taken together, for the first time, the current study demonstrated the anti-inflammatory effects of fargesin on chemically induced IBD might be associated with NF-κB signaling suppression. The findings may contribute to the development of therapies for human IBD by using fargesin or its derivatives.

## 1. Introduction

Inflammatory bowel disease (IBD), which generally refers to ulcerative colitis (UC) and Crohn’s disease (CD), is a progressive inflammatory condition of the gastrointestinal (GI) tract characterized by chronic and relapsing ulceration. IBD is usually associated with severe GI symptoms including abdominal pain, diarrhea, weight loss, and rectal bleeding [1]. Current therapeutics for IBD generally include aminosalicylates, glucocorticosteroids, immunosuppressive drugs, and targeted therapies (e.g., anti-TNF antibody). However, conventional therapeutics cannot prevent adverse effects, toxicity, and complications. For this reason, the development of novel and specific therapies for IBD patients is challenging and in urgent need [2].

Although the exact etiologies of IBD remain unknown, numerous studies have suggested that activation of the nuclear factor kappa B (NF-κB) pathway as well as excessive production of proinflammatory mediators such as nitric oxide (NO), myeloperoxidase (MPO), cyclooxygenase-2 (COX-2), monocyte chemoattractant protein-1 (MCP-1), tumor necrosis factor (TNF)-α, interleukin (IL)-1β, IL-6, etc., play pivotal roles in the pathogenesis of IBD [3]. The transcription factor NF-κB is shown to be a key regulator of proinflammatory mediator genes, and inhibition of NF-κB activation is thought to be a treatment strategy for the control of IBD [1]. NF-κB can be activated by various stimuli, such as proinflammatory cytokines, viral products, and oxidative stress [4]. In the absence of stimuli, NF-κB is sequestered in the cytoplasm by binding to its cytoplasmic inhibitor IκBα. Upon stimulation, IκBα is phosphorylated, and subsequently ubiquitinated and degraded by proteasome, thus allowing the predominant complexes p50/p65 of NF-κB to translocate to the nucleus and activate the transcription of proinflammatory mediator genes [5]. Increased activation of NF-κB and overexpression of NF-κB target genes are present in the gut of IBD patients and murine colitis models [6,7]. Thus, inhibition of the NF-κB signaling pathway has been demonstrated to be an effective approach in controlling mucosal inflammation for IBD treatment [2].

*Magnoliae Flos* (Chinese name: Xin-yi) is a commonly used oriental medicine for the treatment of nasal congestion, allergic rhinitis, sinusitis, and headache. Fargesin, a bioactive lignan isolated from *Magnoliae F*, possesses multiple pharmacological activities, including attenuating oxidative stress, suppressing myocardial apoptosis, improving lipid and glucose metabolism, etc. Lately, fargesin was shown to exert anti-inflammatory effects on THP-1 human monocytic cells through inhibition of NF-κB signaling activation [8]. However, the direct effects of fargesin on intestinal inflammation remain elusive. Thus, we hypothesized that fargesin may block the NF-κB pathway and thereby attenuate murine models of chemically induced IBD.

## 2. Results

### 2.1. In Vivo Study

#### 2.1.1. Effects of Fargesin on Dextran Sulfate Sodium (DSS)-Induced Colitis

**Figure 1:**
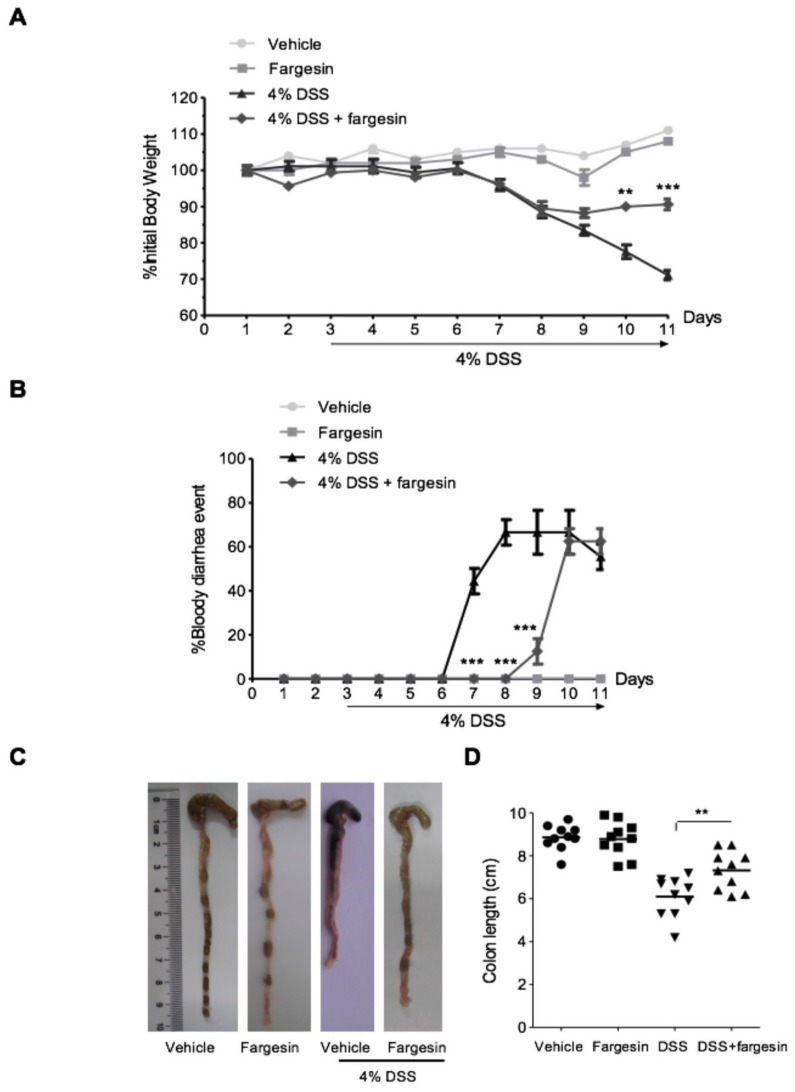
Macroscopic assessment of DSS-induced colitis. (**A**) Body weight changes following DSS induction of colitis. Data were plotted as the percentage of basal body weight. (**B**) The occurrence of bloody diarrhea. Data were plotted as a percentage of the total mice that had bloody diarrhea at different time points of DSS treatment. (**C**) and (**D**) Macroscopic observation and assessment of colon shortening. Values were expressed as the mean ± SD of *n* = 10 mice in each group. ** *p* < 0.01, *** *p* < 0.001 vs. DSS-treated group.

**Figure 2:**
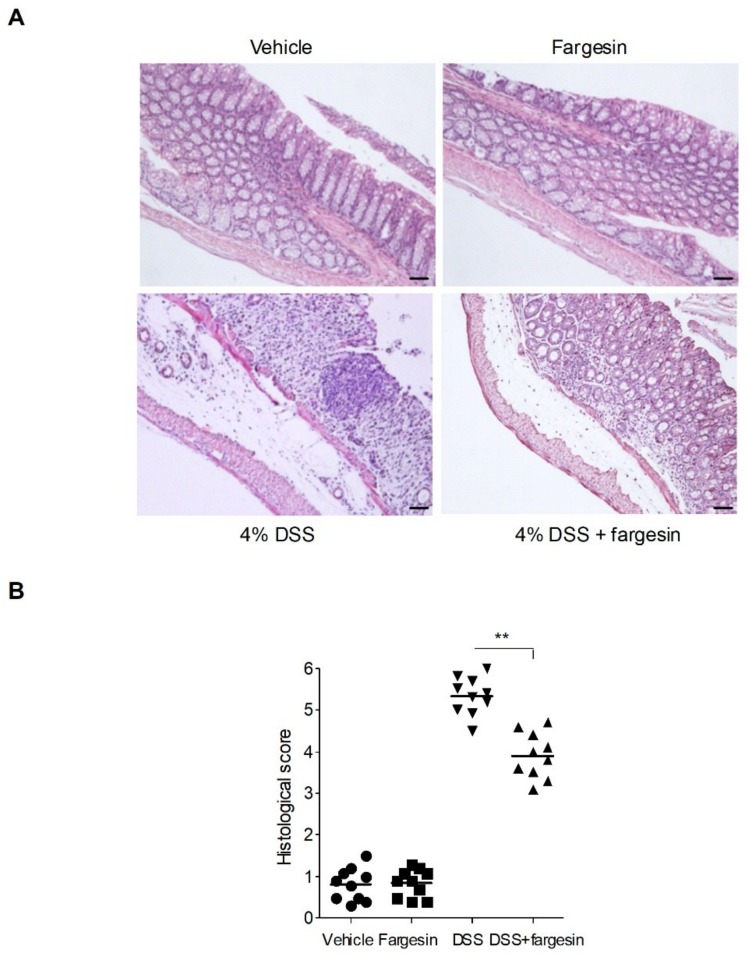
Histological assessment of DSS-induced colitis. Representative hematoxylin and eosin (H&E)-stained colon sections (**A**) and histological score (**B**). Scale bar corresponds to 100 μm. Values were expressed as the mean ± SD of *n* = 10 mice in each group. ** *p* < 0.01 vs. DSS-treated group.

Mice exposed to DSS had a dramatic body weight reduction from day 4 onwards, and oral administration of fargesin showed less body weight loss (Figure 1A). In the DSS-alone treatment group, bloody diarrhea occurred from day 4 till the end of the experiment. Fargesin administration delayed the occurrence and reduced the severity of bloody diarrhea (Figure 1B). The colon shortening is an indication of colitis and therefore can be measured as an inflammatory marker [5]. DSS-alone treatment significantly decreased the length of the colon, and fargesin treatment reversed the reduction in colon length (Figure 1C,D). In the DSS-alone treatment group, DSS caused extensive changes in pathological parameters including edema, crypt distortion, epithelial destruction, ulceration, and inflammatory cell infiltration. The overall histological damage was attenuated by fargesin treatment (Figure 2A,B). Moreover, in the vehicle group and the fargesin group, no weight loss, bloody diarrhea, colon shortening, or histological injury was observed throughout the experiment.

#### 2.1.2. Effects of Fargesin on the Activity of MPO and the Level of Inflammatory Mediators

To evaluate the inflammatory infiltration in the colon in a quantitative manner, MPO activities in the distal colonic tissue were determined. DSS treatment significantly increased the MPO activity as compared with the vehicle-treated group, whereas fargesin treatment effectively decreased MPO activity and the neutrophil infiltration in the injured colon (Table 1).

Because TNF-α has been well characterized as a proinflammatory cytokine that plays a pivotal role in inflammation-related lesions such as IBD, we measured the level of TNF-α in the distal colonic tissue using an enzyme-linked immunosorbent assay (ELISA). A significant elevation of TNF-α content was observed in mice that received DSS-alone treatment compared with the vehicle-treated mice (Table 1). The elevated TNF-α level was significantly decreased in colitis mice treated with fargesin.

**Figure 3:**
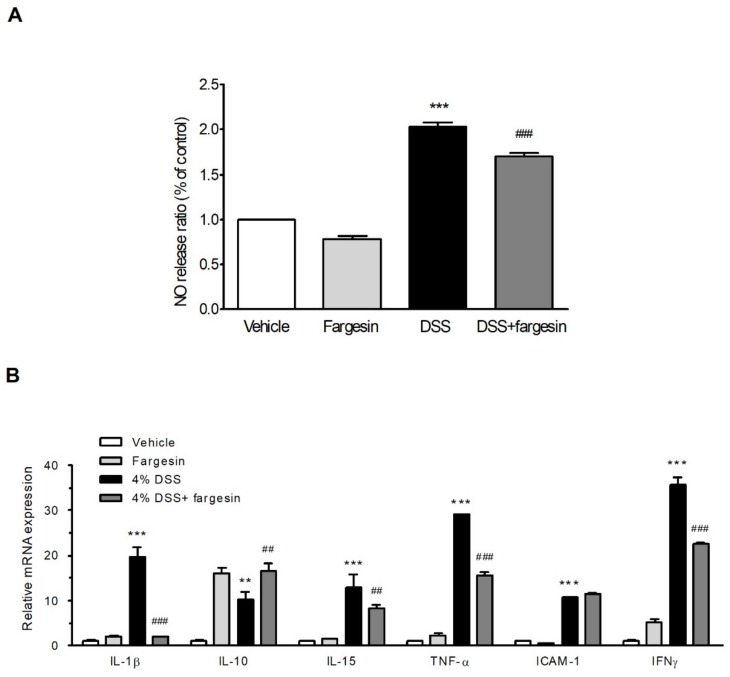
Fargesin downregulated NO production and proinflammatory mediator gene expression in vivo. (**A**) Serum NO level was measured as described in the Methods. Data were expressed as mean ± SD (*n* = 6); (**B**) mRNA expression of proinflammatory genes was determined by qRT-PCR in colon samples. Expression value was normalized to β-actin and each bar represented the mean ± SD of two independent experiments with samples in triplicate. ** *p* < 0.01, *** *p* < 0.001 vs. vehicle-treated group; ^##^ *p* < 0.01, ^###^ *p* < 0.001 vs. DSS-treated group.

Overproduction of NO has been reported to be involved in the pathogenesis of IBD [9]. As expected, we observed an increase in systemic level of NO in serum in the DSS-alone treatment group; however, fargesin treatment decreased the elevated NO level induced by DSS (Figure 3A).

#### 2.1.3. Fargesin Inhibited Pro-Inflammatory Gene Expression in the Colon

To elucidate the potential effects of fargesin on DSS-induced colitis, mRNA levels of proinflammatory mediator genes in the colon were measured by qRT-PCR. Fargesin significantly decreased the expression of proinflammatory cytokines IL-1β, IL-15, TNF-α, and IFNγ and increased the expression of anti-inflammatory cytokine IL-10 in the colon of DSS-alone treatment mice (Figure 3B). However, the DSS-induced upregulation of ICAM-1 expression was not affected by fargesin treatment.

#### 2.1.4. Fargesin Inhibited the Activation of NF-κB in the Colon

**Figure 4:**
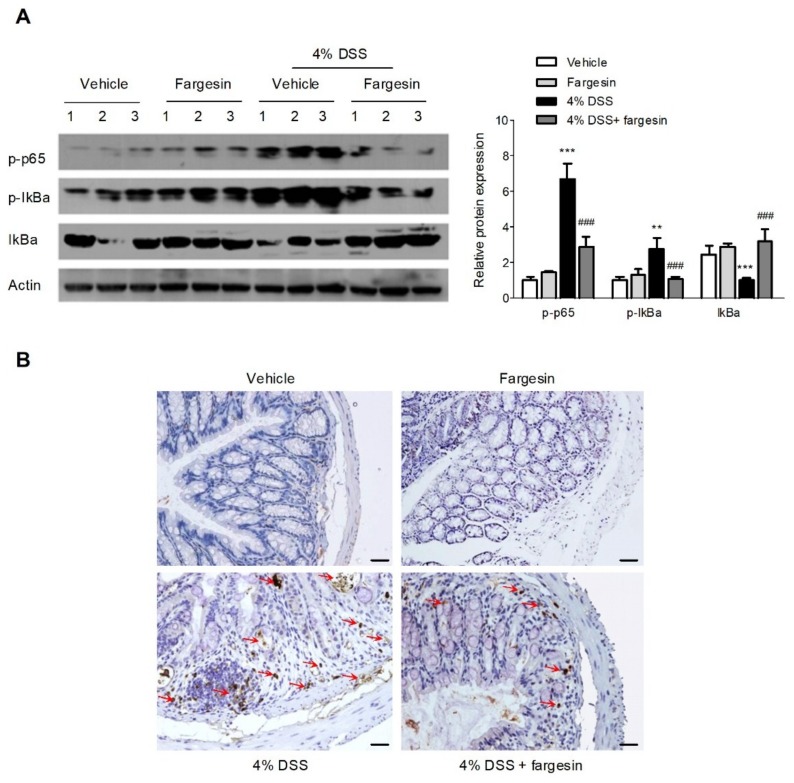
Fargesin inhibited the activation of NF-κB in vivo. (**A**) Protein levels were determined by immunoblotting using p-p65 (1:1000), p-IκBα (1:1000) and IκBα (1:1000) antibodies. One representative experiment is shown. Data were expressed as the mean ± SD of two independent experiments with samples in triplicate. Quantification of the protein expression was performed by densitometric analysis of the blots. (**B**) Representative images of p-p65 immunostaining in colon tissue. Scale bar corresponds to 100 μm ** *p* < 0.01, *** *p* < 0.001 vs. vehicle-treated group; ^###^ *p* < 0.001 vs. DSS-treated group.

NF-κB plays a key part in transcriptional induction of proinflammatory mediator genes, and the activation of NF-κB is thought to be a vital step in the pathogenesis of IBD [1,10]. We detected the effects of fargesin on the activation of NF-κB by Western blot and immunohistochemistry. A significant increase in the phosphorylation of p65 and the phosphorylation/degradation of IκBα was observed in the colon of DSS-alone treatment mice, which was inhibited by fargesin treatment (Figure 4A). Meanwhile, a significant increase in the expression of p-p65 was observed in mucosa epithelial cells of DSS-alone treatment mice; however, administration of fargesin obviously decreased the phosphorylation of p65 in the inflamed mucosa (Figure 4B).

### 2.2. In Vitro Study

#### 2.2.1. Fargesin Inhibited the Nuclear Translocation of p-p65 in RAW264.7 Cells

**Figure 5:**
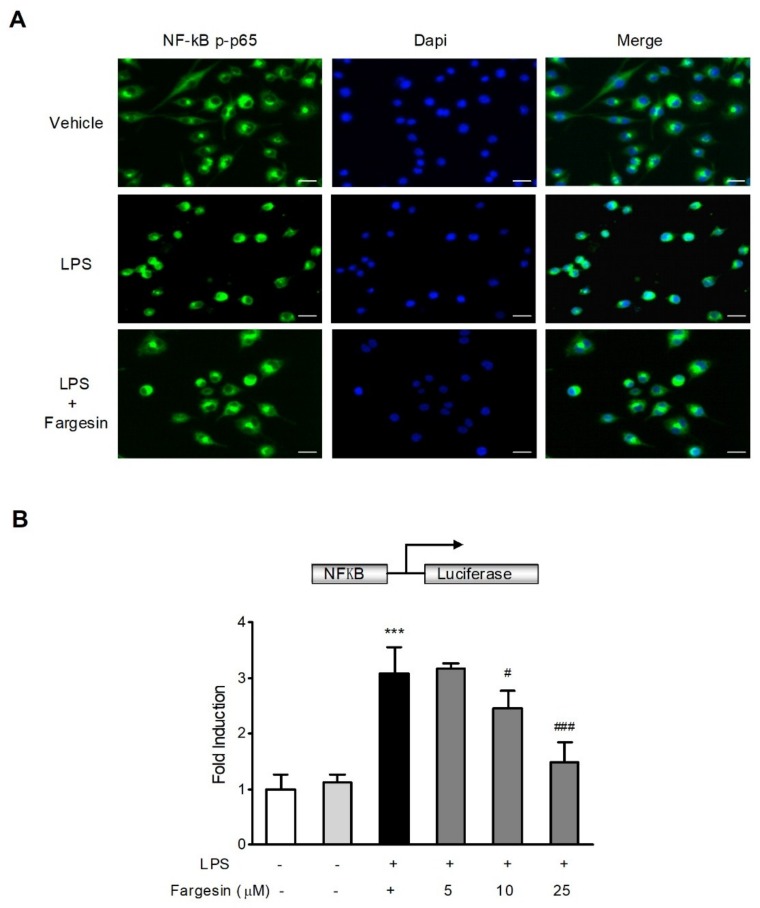
Fargesin blocked the activation of NF-κB in vitro. (**A**) RAW264.7 cells were treated as described in the Methods and p-p65 localization was visualized under a fluorescence microscope. Scale bar corresponds to 200 μm. (**B**) RAW264.7 cells were treated as described in the Methods. NF-κB promoter-driven luciferase activity was determined using a dual luciferase assay system, and values were expressed as the fold induction of the control cells. Data were expressed as mean ± SD of quadruplicates of two independent experiments. *** *p* < 0.01 vs. vehicle-treated cells; ^#^ *p* < 0.05, ^###^ *p* < 0.001 vs. LPS-treated group.

The anti-inflammatory effects of fargesin were further evaluated in RAW267.4 mouse macrophage cells, a widely used cell model for evaluating the in vitro anti-inflammatory effects of compounds [11,12]. As shown in Figure 5A, fargesin obviously inhibited the nuclear translocation of p-p65 in LPS-stimulated RAW267.4 cells.

#### 2.2.2. Fargesin Suppressed NF-κB-Luciferase Activity in RAW264.7 Cells

The NF-κB-luciferase assay was used to assess the effect of fargesin on the inhibition of NF-κB activation in RAW267.4 mouse macrophages cells. LPS caused a significant upregulation in the luciferase activity of the NF-κB reporter, which was reduced by fargesin treatment in a dose dependent manner (Figure 5B).

#### 2.2.3. Fargesin Downregulated Proinflammatory Mediator Gene Expression in RAW264.7 Cells

**Figure 6:**
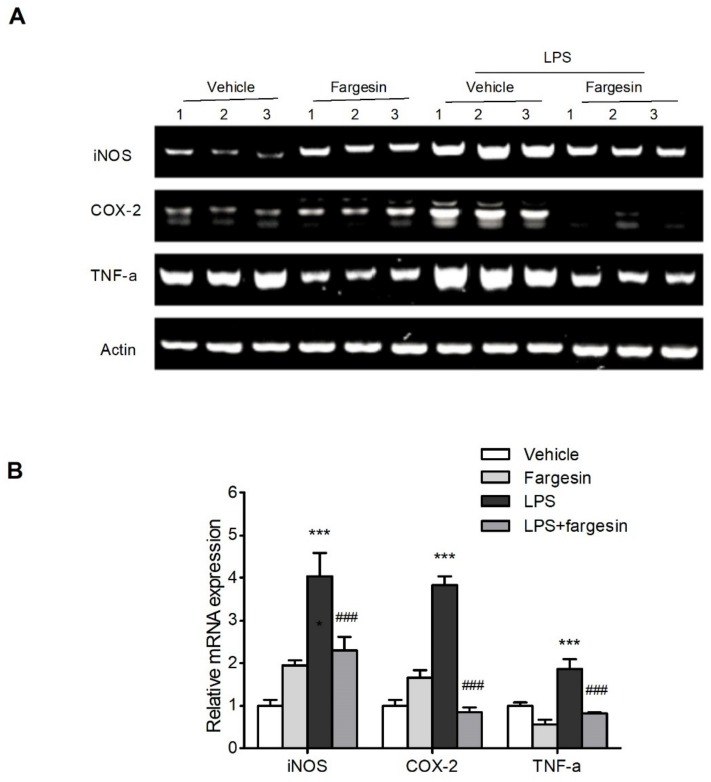
Fargesin downregulated the proinflammatory gene expression in vitro. (**A**) RAW264.7 cells were treated with fargesin (0, 25 μM) for 2 h prior to LPS (2 µg/mL) treatment for an additional 12 h. RNA was extracted as described, and the mRNA expression of proinflammatory genes was carried out by semi-qPCR. The expression was normalized to β-actin. (**B**) Quantification of the mRNA expression was performed by densitometric analysis of the bands. *** *p* < 0.001 vs. vehicle-treated samples; ^###^ *p* < 0.001 vs. LPS-treated group.

The mRNA levels of proinflammatory genes were assessed by semi-qPCR. The increased expression of iNOS, COX-2, and TNF-α in LPS-stimulated RAW264.7 cells was decreased by fargesin treatment (Figure 6).

## 3. Discussion

The addition of DSS to drinking water leads to inflammatory lesions in the colon of rodents characterized by mucosal oedema, infiltration of inflammatory cells, loss of epithelium, and ulceration. Over the past decades, the DSS model of colitis has been perceived as an ideal experimental model of IBD to study its pathological process and therapeutic efficacy [13]. In this study, 4% DSS in drinking water was administrated to induce acute colitis in mice, and the effects of fargesin on colitis were evaluated. The data showed that fargesin ameliorated the disease indexes, such as body weight loss, bloody diarrhea, colon shortening, and histological injury, in the colitis mice. Of note, compared with the vehicle-treated mice, none of the mice receiving fargesin alone exhibited significant morphological or histological changes throughout the study, indicating the relative safety of fargesin treatment.

Although the exact etiology of IBD is not fully disclosed, it has been widely recognized that activation of the NF-κB pathway as well as production of large amounts of proinflammatory cytokines and chemokines are important inducers of mucosal lesions in IBD [14]. Excessive activation of NF-κB has been observed in human IBD and murine IBD model [6,7]. Thus, inhibition of NF-κB is thought to be a treatment strategy for IBD management [2]. In this study, we showed that oral administration of fargesin inhibited IκBα degradation and NF-κB p65 phosphorylation in the inflamed tissue. Meanwhile, fargesin inhibited the nuclear translocation of p-p65 and NF-κB transcriptional activity in LPS-stimulated RAW264.7 cells. These data are consistent with previous observations in which fargesin was shown to be an inhibitor of NF-κB.

MPO is an aperoxidase enzyme secreted from the activated neutrophils. MPO activity, as an indicator of the severity of colitis, is proportional to the level of neutrophil infiltration in the inflammatory tissue [15]. We observed that fargesin reduced MPO activity as well as neutrophil infiltration in the injured colon.

Fargesin also reduced the production and mRNA expression of TNF-α in colonic mucosa of colitis mice. As an important mediator of inflammation, TNF-α plays a critical role in the pathogenesis of IBD [16]. It has been shown that systematic TNF-α level is elevated in IBD patients [17]. Therefore, blockading the generation of TNF-α with varied neutralizing antibodies is indicated to be an effective remedy for IBD, although the side effects, which include serious infection, remain a major concern [17].

Other factors that may be involved in the inflammatory process in IBD include proinflammatory cytokines and chemokines, such as IL-1β, IL-10, IL-15, IFNγ, iNOS, and COX-2 [3]. In our study, fargesin improved some of these parameters in DSS-colitis mice or in RAW264.7 macrophages.

NO is another involved factor in IBD pathogenesis [9]. NO production by iNOS is increased in the mucosal tissue of IBD patients and experimental IBD mouse models [9,18]. Inhibition of NO production has been found to have beneficial effects in easing inflammatory states [18]. In our study, administration of fargesin suppressed the excessive NO production in DSS-colitis mice and downregulated iNOS, COX-2, and TNF-α protein expression in LPS-stimulated RAW264.7 cells. Our findings are consistent with a previous report in which fargesin was shown to reduce NO production and iNOS expression in cytokine-stimulated human respiratory epithelial cells [19].

Currently, there is no effective approach for the treatment of IBD. Therefore, over the past decades, the use of medicinal plants or natural ingredients has become an attractive option in the management of various inflammatory conditions [20,21,22]. In the present study, we determined the attenuated effects of fargesin in DSS-induced colitis and clarified the underlying mechanisms. To our knowledge, for the first time, the present study demonstrated that fargesin attenuated chemically induced IBD via inhibition of NF-κB signaling. Since the relative safety of fargesin management was demonstrated in our study, the findings may contribute to the development of therapies for human IBD by using fargesin or its derivatives.

## 4. Materials and Methods

### 4.1. Materials

DSS (MW 36000-50000 Da) was purchased from MP Biochemical LLC (Solon, OH, USA). Fargesin (Lot No. A0366, HPLC purity ≥ 98%) was kindly provided by the Shanghai R&D Center for Standardization of Traditional Chinese Medicine (Shanghai, China). The following antibodies—NF-κB p65 (#8242), phospho-p65 (#3033), IκBα (#4812), phospho-IκBα (#2859), and β-actin (#4970)—were obtained from Cell Signaling Technology (Danvers, MA, USA). Lipopolysaccharide (LPS), Donkey serum, formaldehyde, paraformaldehyde, Tween-20, sodium carboxymethyl cellulose, DMSO, and ethanol were purchased from Sigma-Aldrich (St. Louis, MO, USA). SYBR Premix ExTaq Mix was from TAKARA Bio (Otsu, Japan). Bovine serum albumin and the protease and phosphatase inhibitor cocktail tablet were obtained from Roche Diagnostics (Mannheim, Germany). Envision-HRP reagent and diaminobenzidine were from DakoCytomation (Carpinteria, CA, USA). 1 × passive lysis buffer and luciferase assay system were purchased from Promega (Madison, WI, USA). Enzyme-linked immunosorbent assay (ELISA) kit for TNF-α was from R&D system (Minneapolis, MN, USA). NO and MPO assay kits were from Nanjing Jiancheng Bioengineering Institute (Nanjing, China). Anti-phospho-NF-κB p65-NLS was from Thermo Scientific (Waltham, MA, USA). The following products were purchased from Invitrogen (Carlsbad, CA, USA): SuperScript III first-strand synthesis system (Thermo Fisher Scientific, Waltham, MA, USA), lipofectamine 2000 transfection reagent (Thermo Fisher Scientific), Fluor 488-conjugated anti-rabbit IgG (A-21206) (Thermo Fisher Scientific), Trizol (QIAGEN, Venlo, The Netherlands), DAPI reagents (Sigma-Aldrich, St. Louis, MI, USA), and Triton X-100 (Sigma-Aldrich).

### 4.2. Animals and Cell Lines

Healthy 8-week-old female C57BL/6 mice (20 ± 2 g) were obtained from the Shanghai Laboratory Animal Center. All mice were housed under standard conditions at Shanghai University of Traditional Chinese Medicine (SHUTCM, 25 ± 2 °C, 60–70% humidity) with a 12 h light/dark cycle. Standard mouse chow pellets and water were allowed for free intake. All experimental procedures were performed in accordance with the guidelines approved by the Animal Ethics Committee of SHUTCM. The RAW264.7 cell line was from American Type Culture Collection (ATCC) and was cultured in Dulbecco’s Modified Eagle’s medium (DMEM) supplemented with 10% foetal bovine serum (FBS) and 1% penicillin-streptomycin.

### 4.3. Experimental Design and Assessment of the Intestinal Inflammatory Process

The experiment lasted for 11 days. C57BL/6 mice were randomly divided into four groups (*n* = 10 mice/group): The vehicle group (mice were given tap water freely and treated with vehicle from day 1 to day 11), Fargesin group (mice were given tap water and treated with 50 mg/kg fargesin from day 1 to day 11), DSS group (mice were given 4% DSS in tap water from day 3 to day 11 and treated with vehicle from day 1 to day 11), and DSS + fargesin group (mice were given 4% DSS in tap water from day 3 to day 11 and treated with 50 mg/kg fargesin from day 1 to day 11). Fargesin was prepared in 0.5% sodium carboxymethyl cellulose. Fargesin dosing was based on previous report and our preliminary studies [23]. The total gavage volume was identical for each group.

Weight, diarrhea, and bloody stool were observed and recorded every day. After sacrifice, the length of the entire colon was measured and photographed. The whole blood was kept at room temperature for 2 h and the supernatant was carefully collected after centrifugation (4 °C, 3000× *g* for 15 min). The serum samples were stored at −80 °C. About 0.5 cm of the distal colon was removed and fixed in 4% formaldehyde overnight Which was then embedded in paraffin and stained with hematoxylin and eosin (H&E). H&E-stained sections of the colon tissue were used for histological analysis. Colonic histological scoring was examined by tissue damage (score 0–3) and inflammatory cell infiltration (score 0–3) using a double-blind method described previously [10]. The distal colon samples were collected and stored at −80 °C for the subsequent analyses.

### 4.4. Western Blot and RNA Analysis

Colon tissues or cultured cells were homogenated and lysed in lysis buffer supplemented with the protease and phosphatase inhibitor cocktail tablet. The lysate was centrifuged (4 °C, 12,000 g, 15 min) and the supernatant was collected. Protein (30 µg) was separated by 10% SDS-PAGE and transferred to nitrocellulose membrane. The blots were blocked with 5% (*w/v*) skim milk for 1 h at room temperature and then incubated with primary antibodies against β-actin, p-p65, IκBα, and p-IκBα overnight. The blots were washed and incubated for 1 h with appropriate HRP-conjugated secondary antibodies at room temperature. Finally, the density of blots was quantified by densitometric analysis.

Total RNA was extracted using a TRIzol reagent according to the manufacturer’s instructions. Complementary DNA (cDNA) was synthesized from 3 μg of total RNA using the SuperScript III Reverse Transcriptase kit. PCR reactions were performed as the manufacturer’s instructions. For semi-qPCR, the annealing temperature was 58 °C. The PCR products were separated by electrophoresis with 2% agarose gels and stained with ethidium bromide. For qPCR, PCR reactions were performed using the reagent of SYBR^®^ Premix Ex TaqMix and quantitatively analyzed by ABI Prism 7900HT Sequence Detection System (Life Technologies, Carlsbad, CA, USA). Results were normalized as the ratio of optimal density relative to β-actin. The primers used are listed in Table 2.

### 4.5. NF-κB Immunohistochemistry and Immunofluorescence

Immunohistochemistry was performed to evaluate phospho-NF-κB p65 expression levels in the paraffin-embedded colon tissue samples. The 4 μm sections were incubated with phospho-NF-κB p65-NLS (1:50) antibody overnight at 4 °C and then incubated with the indicated secondary antibody at room temperature for 1 h. The expression level of phospho-NF-κB p65 was visualized by incubating with diaminobenzidine and counterstaining with hematoxylin.

The immunofluorescence was performed as described previously [24]. Briefly, RAW264.7 cells were exposed to fargesin (25 μM) in advance for 2 h and then incubated with LPS (2 µg/mL) for an additional 12 h. Cells were fixed with 4% paraformaldehyde at room temperature for 10 min. After incubation in a blocking buffer (3% FBS in PBS), the cells were treated with NF-κB p65 antibody overnight at 4 °C, followed by Alexa Fluor 488-conjugated secondary antibody at 37 °C for 60 min in the dark. Nuclei were stained with DAPI (1 µg/mL) for 5 min. Fluorescence photographs were obtained using a fluorescence microscope (Olympus CKX41, Tokyo, Japan).

### 4.6. Determination of TNF-α and NO Levels and MPO Activity

Colon segments (about 0.5 cm from distal colon) were homogenized with ice-cold PBS. The homogenates were centrifuged at 3000 g for 10 min. The levels of TNF-α in the supernatants were detected by a commercially available mouse-specific ELISA kit following the manufacturer’s instruction.

The serum NO level was measured using the NO assay kit as described previously [3]. The results were expressed in the form of the relative release ratio compared with the vehicle group.

MPO activity in colon tissues was measured using a detection kit following the manufacturer’s protocol. The results were expressed as activity units/mg of protein.

### 4.7. NF-κB Luciferase Reporter Assay

RAW264.7 cells were seeded in a 24-well plate at a density of 3 × 10^5^ cells/well. Then, 0.8 µg of pGL4.32 (luc2P/NF-κB-RE/Hygro) vector (Promega, Madison, WI, USA) was transfected into cells using the lipofectamine 2000 reagent as described previously [24]. Cells were exposed to fargesin (5, 10, and 25 μM) in advance for 2 h and then incubated with LPS (2 µg/mL) for an additional 12 h. A dual luciferase reporter assay kit was used to measure luciferase activity. Renilla luciferase was used to normalize the transfection efficiency.

### 4.8. Statistics Analyses

Data were expressed as the mean ± SD. The differences between groups were analyzed by one-way analysis of variance (ANOVA) followed by the least significant difference (LSD) for a post hoc test. Statistical analysis was performed by the SPSS 16.0 software package (IBM Corporation, Armonk, NY, USA). *p*-values < 0.05 (two-sided) were considered significant.

## Figures and Tables

**Table 1:** Effects of fargesin on TNF-α level and MPO activity in colitis mice.

Group	TNF-α (pg/mg pr.)	MPO (U/mg pr.)
Vehicle	1.37 ± 0.05	0.39 ± 0.08
4% DSS	1.86 ± 0.08 ***	0.77 ± 0.27 **
4% DSS + fargesin	1.34 ± 0.18 ^#^	0.37 ± 0.04 ^#^

The level of TNF-α and the activity of MPO were detected according to the specification of the kits. Data were expressed as mean ± SD (*n* = 6). ** *p* < 0.01, *** *p* < 0.001 vs. vehicle group; ^#^ *p* < 0.05 vs. DSS group.

**Table 2:** The list of primers.

S.No.	Gene	Primer Sequence	Annealing Temperature
1	iNOS	F:5′-GGGAATCTTGGAGCGAGTTG-3′ R:5′-GTGAGGGCTTGGCTGAGTGA-3′	58 °C
2	COX-2	F:5′-GAAGTCTTTGGTCTGGTGCCT-3′ R:5′-GCTCCTGCTTGAGTATGTCG-3′	58 °C
3	TNF-α	F: 5′-CGTGGAACTGGCAGAAGAGG-3′ R: 5′-AGACAGAAGAGCGTGGTGGC-3′	58 °C
4	ICAM-1	F: 5′-CGCTGTGCTTTGAGAACTGT-3′ R:5′-AGGTCCTTGCCTACTTGCTG-3′	60 °C
5	IFNγ	F: 5′-AAGTGGCATAGATGTGGAAG-3′ R: 5′-AAGTGGCATAGATGTGGAAG-3′	60 °C
6	IL-1β	F: 5′-GGCTGGACTGTTTCTAATGC-3′ R: 5′-ATGGTTTCTTGTGACCCTGA-3′	60 °C
7	IL-10	F:5′-ACAACATACTGCTAACCGACTC-3′ R: 5′-CACTCTTCACCTGCTCCACT-3′	60 °C
8	IL-15	F: 5′-CAGAATGGGAGGTGGTAGTGC-3′ R: 5′-AAGAGTGGCTGGACAGAAGG-3′	60 °C
9	β-actin	F:5′-CAGCCTTCCTTCTTGGGTAT-3′ R: 5′-TGGCATAGAGGTCTTTACGG-3′	58 °C/60 °C
